# Crossing the Borders: An Integrated Approach to Myeloproliferative Neoplasms and Mastocytoses

**DOI:** 10.3390/cancers13071492

**Published:** 2021-03-24

**Authors:** Marco Pizzi

**Affiliations:** Surgical Pathology and Cytopathology Unit, Department of Medicine-DIMED, University of Padua, 35121 Padua, Italy; marco.pizzi.1@unipd.it

Since the first description of Chronic Myeloid Leukemia (CML) as a “suppuration of the blood” in 1845 [1], myeloproliferative disorders have revealed their challenging and disguising nature. Throughout the 20th century (especially in the last decades), major advances have been made on the biology and on the clinical-pathological features of these entities. These acquisitions have led to the current WHO Classification of Hematopoietic and Lymphoid Tumors [2] and to a deep molecular characterization of Myeloproliferative Neoplasms (MPNs) and related tumors [3].

MPNs can be broadly classified into the following: (i) CML, *BCR-ABL1*-positive (bearing the Philadelphia chromosome in most cases); (ii) classical Philadelphia-negative MPNs (Essential Thrombocythaemia, ET; Polycythaemia Vera, PV; Primary Myelofibrosis, PMF); (iii) non-classical Philadelphia-negative MPNs (Chronic Neutrophilic Leukemia, CNL; Chronic Eosinophilic Leukemia not otherwise specified, CEL NOS); and (iv) MPN, unclassifiable. Mastocytosis (formerly considered within the spectrum of MPNs) is now regarded as a distinct disease category due to its unique pathogenic and biological features [2].

This classification relies on clinical-histological presentations as well as on recurrent molecular derangements. By definition, CML harbors *BCR-ABL1* fusions, while Philadelphia-negative MPNs have more variable genetic profiles. Most ETs and PMFs disclose driver mutations in *JAK2* (V617F), *CALR* or *MPL*, and virtually all PVs are mutated in *JAK2* (V617F or exon 12). CNLs are frequently associated with *CSF3R* mutations, CEL NOS lacks specific genetic/cytogenetic profiles, and mastocytoses show activating mutations of *KIT* (D816V and variants) [2]. Besides these changes, several mutations in epigenetic/DNA methylation regulators (e.g., *TET2*, *DNMT3A, IDH1/IDH2, EZH2*), splicing factors (e.g., *SF3B1, SRSF2, U2AF1*), signaling pathways (e.g., *NF1*, *NRAS, KRAS, FLT3*), tumor suppressors (*TP53*) and transcription factors (e.g., *ETV6, RUNX1)* are reported across MPNs and variably correlate with outcome [3,4].

This well-defined pathogenic landscape may provide the wrong impression that MPNs are just a matter of laboratory findings and gene mutations. In fact, areas of uncertainty remain, and clinical practice reminds us that the boundary between entities is not always so clear. Biologically distinct disorders may indeed present similar clinical-laboratory findings and overlapping mutations [2]. Some patterns of disease evolution mimic unrelated myeloid disorders [5,6], and unusual associations suggest a common origin for apparently distinct hematological neoplasms [7].

To cope with this complexity, pathologists and clinicians need to cross the borders of specialties and subspecialties. Trustable diagnoses and patient management require the integration of multiple competencies, approaching the problem from different perspectives. Crossing the borders in MPNs and mastocytoses also means matching clinical-pathological data with basic science studies, which are rapidly expanding our understanding of these tumors. Such integration will allow solving most ambiguities in difficult cases, always within the frame of the WHO Classification.

One of the mentors of my training years used to say that the WHO “Blue Books” are like the law and that pathologists and clinicians are like judges, who make diagnoses by applying this law. In the same way that judges determine sentences by considering the peculiarities of each case, physicians should approach blood disorders by integrating the WHO criteria and the specific features of cases they are confronted with. This is made possible only by a thorough understanding of the biological and clinical complexity of hematopoietic tumors.

To pay tribute to such view, this Special Issue will present a comprehensive and up-to-date picture of the multifaceted aspects of MPNs and mastocytoses. All of this will hopefully contribute to shed light on these fascinating neoplasms, supporting their diagnosis and clinical management.

## List of Abbreviations:

CEL, NOS	Chronic Eosinophilic Leukemia, not otherwise specified
CML	Chronic Myeloid Leukemia
CNL	Chronic Neutrophilic Leukemia
ET	Essential Thrombocythaemia
MPN	Myeloproliferative neoplasm
PMF	Primary Myelofibrosis
PV	Polycythaemia Vera
